# A large-scale dataset for analysing remote working in urban and rural areas across Europe

**DOI:** 10.1038/s41597-025-05972-z

**Published:** 2025-10-23

**Authors:** Katharina Fellnhofer, Margarita Angelidou, Johannes Flacke, Lisa Fontanella, Mandy Fransz, Özge Karanfil, Eirini Kelmali, Sibel Kiran, Pavlos Kolias, Vidit Kundu, Konstantina Mataftsi, Thomas Mone, Greta Nasi, Hakan S. Orer, Marilena Papageorgiou, Panagiotis Papanikolaou, Karin Pfeffer, Theodora Istoriou, Dimitra Plastara, Kelly Pasmatzi, Georgia Pozoukidou, Alexandra Prodromidou, Elli Roma-Athanasiadou, Sibel Sakarya, Giovanni Oscar Serafini, Efstratios Stylianidis, Surucu Huseyin Selçuk, Ioannis Tavantzis, Nikolas Thomopoulos, Zoi-Eirini Tsifodimou, Christos Menelaos Vlemmas, Tracy Xu, İlker Kayı

**Affiliations:** 1 Research and Innovation Management GmbH, Neumarkt an der Ybbs, Austria; 2 ETH Zürich, Zürich, Switzerland; 3 Aristotle University of Thessaloniki, Thessaloniki, Greece; 4 Q-PLAN International Advisors P.C, Thessaloniki, Greece; 5 University of Twente, Enschede, The Netherlands; 6 SDA Bocconi School of Management, Milano, Italy; 7 WFA Collaborative OÜ, Tallinn, Estonia; 8 Koc University, Istanbul, Türkiye; 9 MIT Sloan School of Management, Cambridge, MA 02142 USA; 10 South-East European Research Centre-CITY College, University of York Europe Campus, Thessaloniki, Greece; 11 White Research SRL, Brussels, Belgium; 12 Bocconi University, Milano, Italy; 13 Arx.Net SA, Thessaloniki, Greece; 14 Surrey Business School, Future of Work Research Centre, University of Surrey, Guildford, UK

**Keywords:** Economics, Geography, Business

## Abstract

This Data Descriptor presents the collected data on remote work among urban and rural workers, emphasizing differences in perceived flexibility, adaptability, preferences, career impacts, well-being, and productivity. The survey, conducted via Prolific between July and August 2024, captured data from 20,013 participants globally with European nationality, offering insights into the benefits and challenges faced by remote workers. Beyond its initial scope, the dataset can be reused to support a wide range of future studies and policy development initiatives. For instance, researchers can utilize this dataset to explore trends in work-life balance, productivity, and urban policy and planning related to remote work. Additionally, urban planners and policymakers can leverage the data to inform strategies for urban and regional development, infrastructure forecasting, planning, and community support in diverse geographic areas. The dataset’s granularity on socio-economic factors makes it an invaluable resource for developing targeted policies that address urban-rural disparities and foster sustainable remote working arrangements across Europe.

## Background & Summary

Remote work, first conceptualised in the 1970s^1^ has recently attracted the interest of many individuals, not due to its novelty but because of the rapid escalation in the incidence of remote or hybrid work in the wake of the COVID-19 pandemic. As a means of engaging with the labour market, remote work holds great potential to advance European Union’s territorial cohesion across social, economic and environmental dimensions, while aligning with the European Regional Development Fund’s 2021–2027 priorities. It enhances competitiveness by allowing businesses to become more agile and tap into a broader talent pool^2,3^, fosters inclusion^4^ and higher employment rates^5^ boosting local economies^6^ and promotes greater mobility through flexible living and working arrangements^7^ making Europe more connected, reducing the need for commuting, and contributing to a greener Europe^8^.

Remote work, particularly after the COVID-19 pandemic, has emerged as a pivotal strategy for mitigating the urban-rural divide, presenting a viable countermeasure to rural depopulation across Europe (Eurofound^9^, p. 2). It is thus logical that remote work receives increasingly attention as a mode of labour market engagement. This attention is not driven by the novelty of the concept, but rather the increasing availability of suitable digital tools and internet accessibility. The adoption of remote work during the pandemic exhibited significant variability across European countries, as well as among different regions and degrees of urbanization. Prior research concluded that teleworkable employment was more prevalent in urban rather than rural areas^10^.

Despite the uneven distribution of remote work and the uneven possibilities to conduct remote work (e.g., differences in terms of digital accessibility) across urban and rural areas, as well as amongst countries^11,12^, its adoption has yielded significant advantages. It has enabled people to relocate to more affordable areas and provided rural residents access to urban labour markets. This dynamic has garnered the attention of European policymakers, who are increasingly focused on assessing the prevenance of remote work across European regions and understanding the drivers behind these disparities of uneven remote work, with a view to promote telework in underrepresented areas, thereby maximizing its potential benefits (European Foundation for the Improvement of Living and Working Conditions^10^, p. 13).

Moreover, remote work has shown significant implications for both urban mobility and workplace dynamics. A study analyzing U.S. data from April 2020 to October 2022 found that even a small shift away from on-site work leads to notable declines in vehicle miles travelled and transit ridership, suggesting that remote work can substantially reduce transportation-related carbon emissions while also decreasing transit fare revenue^13^. Complementing these environmental insights, a randomized control trial in a Chinese tech company revealed that hybrid remote work improves employee satisfaction and retention without harming performance. Notably, quit rates dropped by one-third, especially among women, non-managers, and long-distance commuters. Performance reviews, promotions, and productivity remained unaffected, and managerial attitudes toward remote work shifted positively^14^. Together, these findings highlight that remote and hybrid work models can offer sustainability and workforce stability benefits, without compromising organizational productivity or long-term employee advancement.

The dataset documented in this article was collected as part of the European Commission-funded R-Map project (grant agreement 101132497), which aims to understand, predict, and propose solutions and reformulate it into policy-recommendations for the impacts of remote working arrangements on the spatial, economic, and social dimensions of the urban-rural divide in Europe. The research is grounded in the premise that bridging the urban-rural divide is a persistent challenge in Europe, with three distinct dimensions: spatial, economic and societal. Remote working arrangements are recognized to have an impact on all three dimensions and consequently the urban-rural divide as a complex and multifaceted phenomenon. To better understand the drivers, enablers and barriers influencing the adoption of remote work, the project developed and conducted a large-scale survey covering multiple European countries. Informed by existing evidence, the data collected focuses on key factors such as employee preferences, perceptions on how remote work affects various aspects of their personal and professional lives and the influence of its modality (e.g., fully remote vs. hybrid) on relocation decisions. These aspects are examined in relation to location-specific factors, such as amenities, facilities and infrastructure as well as subjective employee perceptions. Comprising 24 items, the collected data is broadly categorized into three thematic areas:

1. **Perceptions and Experience of Remote Work**. This thematic area focuses on individual attributes, preferences and experiences with remote work arrangements. It explores aspects such as flexibility, personal impact, work life balance and productivity. The theme includes the examination of the type of remote work (i.e. fully remote or hybrid arrangements) and location where work is conducted (i.e. traditional office, third place or coworking space). Collecting data on the number of hours employees spend working remotely, provides a comprehensive view of the temporal distribution of remote work across Europe. Additionally, examining the location where remote work is conducted is particularly important, given the growing focus on coworking spaces as a hybrid environment that foster connections between individual and their community (see Mariotti and Manzini-Ceinar^15^). This emphasis is further underscored by the relative scarcity of systematic data on rural coworking and the ability of coworking spaces to stimulate socioeconomic revitalization in rural areas^16^. The theme also addresses perceptions of the benefits and drawbacks of remote work. This includes exploring attitudes towards flexibility in remote work locations, the impact on personal life, well-being, work-life balance, productivity and other perceived challenges.
2. **Spatial Factors, Relocation Practices and Mobility Patterns**. This dimension examines the spatial, social and economic factors that either facilitate or hinder the adoption of remote work as well as the associated relocation practices and mobility patterns. It is evaluated through three key items. The first evaluates proximity to and perceived importance of amenities, such as schools, healthcare and recreational spaces. The second assesses the perceived significance of numerous social, economic and infrastructural factors as drivers of relocation decisions. The third focus on both daily and long-term mobility patterns analysing their potential as outcome of remote work, particularly within the context of the flexibility remote work provides. These items collectively highlight disparities between urban and rural areas, as well as among different urban typologies (urban, suburban, exurban), shedding light on the ways in which remote work influences daily routines and lifestyle choices^17,18^.
  In specific, proximity to key amenities such as schools, groceries, healthcare facilities, etc., plays a critical role in determining the attractiveness of remote work, influencing work life balance, productivity and overall satisfaction^8^. Furthermore, remote work eliminates the need for the daily commute, offering individuals greater flexibility in selecting their place of residence. Relocation decisions are influenced by various factors, such as family ties, affordability, transportation accessibility, proximity to nature, etc.^8^. These emerging patterns of residential location choices are anticipated to have a measurable impact on the urban-rural divide^19^.
  This study explores long-term and daily mobility as potential outcomes of remote work, particularly in relation to the flexibility it offers. Daily mobility is assessed through the duration and modes of transport used to reach work locations, while long-term mobility is examined by analysing relocation practices. The analysis includes the spatial dimensions of these relocations, such as intraregional, interregional, and cross-border movements. Additionally, mobility patterns are categorized along the urban-rural continuum, distinguishing between urban-to-urban, rural-to-urban, urban-to-rural, and rural-to-rural relocations. Thus, we used an approximate location to determine where people live.
3. **Demographics** and **Employment Context**. Demographic information such as age, gender, educational background, professional experience, employment sector and status of remote workers are used to contextualise responses within the broader socioeconomic contexts.

Overall, the rapid evolution of remote work, amplified by COVID 19 pandemic, has underscored its transformative potential in advancing European Union’s territorial cohesion goals. Despite disparities in adoption between urban and rural areas, remote work presents opportunities to address systematic challenges such as rural depopulation and uneven access to labour markets. In this study, we primarily focus on the adult working population; however, for future research, we propose expanding the scope to include access to education—particularly remote education—as a means to bridge the urban-rural divide. Policy makers and researchers alike are keen to explore implications for mobility patters, residential choices, and socioeconomic revitalization, particularly in underrepresented regions, prompting policy considerations on a national and supranational level. Against this backdrop we collected data to provide a comprehensive framework for examining the drivers, enablers, and barriers to remote work adoption.

## Methods

The study received ethical approval from the German Association for Experimental Economic Research e.V. (No. KEF58hmh, https://gfew.de/ethik/KEF58hmh). We obtained electronic informed consent from all participants. Data collection occurred via Prolific (https://www.prolific.com, Prolific, 2025) in July and August 2024, involving 20,013 participants with complete data entries, all residing in Europe or holding European nationality. Altogether data was collected from 21,312 individuals including missing data. All participants answered the same set of survey questions, which were translated into Greek, Dutch, Portuguese, German, and Turkish. The survey, designed using LimeSurvey (https://www.limesurvey.org, LimeSurvey, 2025), was distributed through Prolific. The resulting dataset, provided in an Excel file, includes all survey items and responses.

### Methodological approach

The questionnaire was designed to explore the socioeconomic and spatial effects of remote working arrangements across Europe, with a focus on understanding working and living conditions, employment dynamics, and quality of life. Its development followed a structured, multi-step process:

1. **Literature Review**. Initial desk research examined existing studies and reports on the implications of remote work, including effects on work-life balance, occupational health, employment formats, spatial implications and socioeconomic outcomes.
2. **Co-Creation**. In line with recommendations from the literature (e.g., Dillman *et al*.^20^) the questionnaire items were formulated collaboratively among 27 experts in socioeconomic research, urban studies, and occupational health. Statements employed Likert scales and were designed to present balanced perspectives to minimize response biases. Spatial and demographic dimensions were also incorporated.
3. **Translation**. We translated the survey into five languages—Turkish, Greek, German, Portuguese, and Dutch—with the assistance of ChatGPT (https://chat.openai.com, OpenAI, 2025). The translations were refined through a back-and-forth process within the author team to ensure comprehension, clarity, accuracy and consistency.
4. **Ethical and Legal Considerations**. To ensure compliance with GDPR standards and safeguard participant anonymity, the survey excluded personally identifiable information while retaining georeferenced data for regional and spatial analysis. Although we initially requested information on the municipalities of participants’ work and residence to determine whether their location was rural or urban, this data was later deleted. We obtained electronic informed consent from all participants for the publication of anonymized data.
5. **Pilot Testing**. A trial run with 45 respondents was conducted to evaluate question clarity, identify technical issues, and refine the instrument based on participant feedback.

The finalized questionnaire comprises 24 structured questions, corresponding to the three thematic areas outlined in the first section of this paper, and further detailed in the section on Variables.

### Data collection

Data were collected in July and August 2024 using LimeSurvey as the survey platform and Prolific for participant recruitment. Participant recruitment on Prolific was based on the following criteria: (1) Geographical Representation: Distribution across countries in the European Union (including the United Kingdom, Switzerland, and Turkey) proportionate to their 2023 populations, (2) Gender Parity: Equal gender distribution within each country, and (3) Age Targeting: Targeting the mean age per country through simple random sampling. 21,312 responses were collected during this period whereas 20,013 provide complete dataset. A total of 299 responses were removed due to incompleteness. Participants responded to identical survey items in their preferred language.

### Variables

This section presents the individual survey questions into the three initial thematic areas: (i) perceptions and experience of remote work, (ii) spatial factors, relocation practices and mobility patterns and (iii) demographics and employment context.

For the first thematic area, perceptions and experience of remote work, 24 questions provides insights to eight variables. We used a Likert scale from strongly disagree (1) to strongly agree (7) to measure each individual’s level of agreement with the following statements:

1. It is important for me to have the flexibility to choose my work location;
2. Remote work positively impacts my personal life;
3. It is important for me to adjust my work schedule based on personal circumstances;
4. I prefer remote work over in-office work;
5. Remote work negatively impacts my career advancement (promotion, recognition, skill development);
6. I find it difficult to maintain a healthy work-life balance while working remotely;
7. I find remote work more challenging than in-office work (e.g., isolation, more distractions, time zone differences);
8. I feel less productive while working remotely (more distractions, no focus, bad time management, etc.).

The answers to these statements provided a holistic understanding of the needs and the perceptions of individual regarding the versatile implications on flexibility, remote working benefits, adjustment to personal circumstances, remote work preferences, negative impact, well-being, challenges, and productivity.

For the second and third themes of the questionnaire, we employ mixed response formats tailored to each variable’s context and dimensions. These include yes/no questions to assess binary outcomes and ask more questions, multiple-choice questions to capture diverse options, single-choice questions for prioritization or preference, and importance ratings to evaluate the significance attributed to specific factors. This approach ensured flexibility and precision in capturing the multifaceted dimensions of the phenomenon across the following variables:

1. On average, what amount of time of your weekly work schedule do you perform remotely/ in the office?
2. When working remotely, do you currently have access to the following amenities within a 15-minute walk;
3. Have you changed your place of living and/or working due to favourable remote working arrangements since 2020?
4. Name the 5 most important reasons that led to your place of living relocation;
5. How much time do you spend on commuting to work and how do you usually commute to work.

For the demographics, the variables included, (1) gender, (2) age, (3) time spend in education, (4) time spend in professional working, (5) place of residence, (6) place of work, (7) industry sector, and (8) employment status.

Following is a description of the individual survey questions per theme.

#### Perceptions and experience of remote work

##### Perceived flexibility

To explore the concept of perceived flexibility, the survey included the item “It is important for me to have the flexibility to choose my work location.”. Workplace flexibility refers to “the ability of workers to make choices influencing when, where, and for how long they engage in work-related tasks.” (Hill *et al*.^21^, p. 152). Such flexibility embodies a reciprocal trust and respect between employer and employee, or between supervisors and their reports, and relies on a supportive organisational culture that grants individuals’ substantial autonomy over their roles and working conditions^22^. Hence, perceived flexibility links to notions of overall employee job satisfaction, engagement and, ultimately, retention^23^. This is also particularly true in relation to the young generation, which prizes workforce flexibility much more than older generations leading to a remarkable positive impact on employee engagement^24^. Studies have shown that in recent years flexibility in relation to location and work schedule has been increasingly considered to be two of the main parameters that define the quality of employment, alongside career opportunities and income. Flexibility has been found to correlate positively with job satisfaction and organizational commitment while at the same time, it correlates negatively with exit intentions.

Marx *et al*.^25^, for instance, confirmed in their study that providing employees with the choice of home-based teleworking correlated negatively with voluntary employee exit. Additional studies provide further evidence of applicant attraction when time/spatial flexibility is offered^26^. When it comes to overall job satisfaction, Bjärntoft *et al*.^27^ identified in their study that perceived flexibility positively associated with work-life balance and in many cases mitigated the negative effects of remote working, such as over-commitment to work and high job demands.

Transitioning from the broader benefits, challenges, and implications of remote work to the specific dynamics of participants’ working arrangements, respondents were asked to quantify their weekly division of hours between remote and in-office work. Subsequently, participants selected their preferred location for remote work from three options: (1) the home, (2) a third place (libraries, cafes, community buildings, etc.) and (3) a co-working space. For those preferring third places or co-working spaces, they were asked to indicate by choosing yes or no if their preference was linked to (1) the availability of car parking, (2) the accessibility via public transport and (3) its proximity within walking or cycling distance from home. This section of questions aimed to provide an understanding of participants’ remote work arrangements.

##### Remote work benefits

The benefits of remote work were examined through the survey item “Remote work positively impacts my personal life.”. The advantages of remote work are intrinsically linked to work-life balance and well-being, which is a multilayered topic. Remote work has had both positive and negative correlations with work-life balance, especially in relation to healthy and sustainable boundaries, burnout, impact on retention and productivity. For example, Ferreira and Gomes^28^ conducted a study across European countries during the COVID-19 pandemic, which demonstrated a strong association between individual and organizational resources and the achievement of work–life balance. Conversely, a study by Buonomo *et al*.^29^ found no significant direct or indirect link between leader support and well-being but rather highlighted the importance of strong social connections as a major factor in employee well-being. Additionally, Sandoval-Reyes^30^ introduced another dimension to work-life balance by discussing gender asymmetries in married couples, such as uneven distribution of childcare and home care responsibilities, showcasing an example of inequality that can determine the impact of remote work on employees’ personal life.

##### Adjustment to personal circumstances

The ability to adapt work schedules to personal circumstances was assessed through the survey item “It is important for me to adjust my work schedule based on personal circumstances.”. This concept is closely related to work-life balance, flexibility and autonomy, all of which play a pivotal role in shaping modern workplace dynamics. Research underscores the value of non-monetary policies, such as flexible working hours, in prompting work life balance. The importance of flexibility in remote work is furthered highlighted by calls to include more ‘inclusive flexibility’ in workplace policies^31^. For instance, remote working options could potentially become more inclusive for groups of people with chronic illnesses, who would otherwise be less employable. To this end, a study by Vanajan *et al*.^32^ on older workers experiencing long-term health issues showed that flexible work hours have a positive impact on their ability to work. Additionally, remote work policies could help companies overcome regional talent acquisition challenges as it enlarges the pool of potential employees by offering time-flexible location-independent employment^33^.

##### Remote work preferences

The preference for remote work over in-office arrangements was assessed through the survey item “I prefer remote work over in-office work.”. This concept is intrinsically linked to autonomy, time and spatial flexibility and communication. Research on time and spatial flexibility has yield mixed findings regarding its impacts on work-life balance and performance. Metselaar *et al*.^34^ showcased that autonomy, i.e. the freedom of the employee to determine when and where to work, has a direct positive correlation with employee performance. Similarly, Wheatley *et al*.^31^ emphasized the importance of ‘inclusive flexibility’ and ‘responsible autonomy’ in organizational policies. These approaches advocate for tailoring remote work policies on the individual needs of the employees, while employees would be accountable for the responsible use of their work autonomy.

Employee preferences for remote or in-office work, particularly within hybrid work models, often depend on the type of tasks involved. For instance, tasks requiring deep concentration might be more suited to remote work as opposed to those requiring communication. Jämsen *et al*.^35^ highlighted that effective communication can be challenging in remote settings, making employees in communication-based tasks, such as managers, more likely to prefer in-office environments. These findings underline that when autonomy can be exercised, discrepancies in preferences can be found across different industries or company roles.

##### Negative impact on career advancement

The potential negative impact of remote work on career advancement was assessed through the survey item “Remote work negatively impacts my career advancement (promotion, recognition, skill development).”^36^. Τhis issue has been widely discussed in media articles of general interest, in public forums and groups dedicated to remote workers, often framed with the “out of sight, out of mind” narrative (Countouris & De Stefano^37^, p. 147). While it may be premature to draw definitive conclusions, several studies in relevant literature have begun to examine this issue. For example, Emanuel *et al*.^38^ investigated the effect of physical proximity among software engineers at a Fortune 500 online retailer. Their findings indicate that proximity encourages junior engineers to seek and receive more feedback and ask more follow-up questions, while female engineers participate more actively in feedback exchanges^38^. The impact of proximity does not immediately translate to career advancement^38^. Initially, junior engineers experience modest opportunities for pay raises. However, over time, the human capital developed through close mentorship becomes evident, often resulting in more substantial pay increases and, in many cases, leading junior engineers to higher-paying positions in other companies^38^. This suggests that close collaboration with colleagues, particularly mentors, has a direct effect on the career advancement of junior employees. In contrast physical distance can constrain access to mentorship opportunities and skill-building, especially for female employees^38^. In contrast, as the trend towards remote and hybrid work arrangements grows, certain companies, such as X (formerly Twitter) under Elon Musk’s leadership, have mandated a return to office-based work^39^. This shift has prompted researchers to advocate for the abandonment of traditional, control-oriented managerial strategies in favour of strategies that align with evolving work dynamics and prioritization of employee autonomy and adaptability^40^.

##### Impact on well-being

The challenges of maintaining work-life balance in a remote work setting were examined through the survey item “I find it difficult to maintain a healthy work-life balance while working remotely.”^36^. While remote work is positively correlated with job satisfaction and overall job-related well-being, research indicates that remote workers often struggle to detach from their duties after working hours^41^. This inability to “disconnect” highlights a crucial issue in maintaining work-life balance for remote employees. Drawing on Social Exchange Theory, Felstead and Henseke^41^, suggest that remote workers perceive the flexibility offered by remote work as being accompanied by certain trade-offs. Additionally, Border Theory challenges the blurring boundaries between work and home life, as the end of work hours becomes less defined.

Grant, Wallace, and Spurgeon^42^ further discuss the difficulty employees face in setting boundaries. However, some participants expressed satisfaction with the blurred boundaries, as they could work during off-hours, allowing them to work outside conventional hours and allocate typical work hours in other activities. This highlights the need for adaptive behaviors among remote workers to manage these blurred boundaries effectively^42^. Buonomo *et al*.^29^, using the Conservation of Resources (COR) theory—which supports individuals’ effort to protect existing resources while seeking to acquire additional valuable resources—emphasize the importance of a strong support network among colleagues in maintaining work-life balance. They find that remote workers who view their job as a valuable resource and who receive support from their colleagues experience better work-life balance. This relationship is further enhanced by job satisfaction, which acts as a stabilizing resource for remote employees. Additionally, Buonomo *et al*.^29^ underscore the importance of managerial communication with remote employees, addressing emotional well-being, time management, stress, and work-life balance^29^. Proactive organizational support systems help safeguarding the mental health and overall well-being of remote workers, thereby fostering a healthier and more balanced remote work environment^29^.

##### Challenges associated with remote work

The challenges of remote work were assessed through the survey item “I find remote work more challenging than in-office work (e.g., isolation, more distractions, time zone differences).”^36^. Remote work presents a range of challenges that affect both personal and professional aspects of employees’ lives. While these challenges often lead remote workers to seek opportunities for self and professional growth, they can also foster feelings of isolation, both personally and socially^43^. During the COVID-19 pandemic, many employees struggled to adapt to the new remote work reality, facing increased distractions and a disrupted sense of routine outside the traditional workplace environment, which further destabilized their work-life balance^44^. Using the Conservation of Resources (COR) theory and the challenge-hindrance framework, Franken *et al*.^44^ explored these experiences. According to COR theory, individuals strive to preserve resources they deem valuable. The challenge-hindrance framework further suggests that employees embrace manageable challenges but, when faced with overwhelming obstacles, may conserve their resources by disengaging from tasks they view as unmanageable (Cavanaugh *et al*.^45^, in Franken *et al*.^44^). Franken *et al*.^44^ concluded that a home environment, if not well-suited as a workspace, can lead to resource loss, particularly when it lacks ergonomic adaptations. Additionally, inadequate technological support can exacerbate this sense of resource depletion, creating a challenging environment for remote workers. Oleniuch^46^ also highlights differences between experienced and inexperienced remote workers. Inexperienced remote workers are more likely to report a decline in living comfort, while experienced remote workers frequently mention issues of isolation and the need for self-discipline. Overall, the literature suggests that remote work requires a diverse skill set to effectively manage its unique challenges, emphasizing adaptability, resource management, and self-motivation.

##### Productivity perceptions

We asked following item “I feel less productive while working remotely (more distractions, no focus, bad time management, etc.).”^36^. Research indicates that remote work can enhance productivity and employee satisfaction when accompanied by appropriate solutions to its challenges.

Recent research highlights the dual nature of remote work in shaping employee motivation and productivity. According to Safri *et al*.^47^, remote work arrangements offer several benefits, including reduced commuting time, fewer unnecessary meetings, improved focus in a home environment, and greater autonomy—factors that can enhance employee motivation and overall output. However, their study also identifies key challenges such as technical issues, diminished concentration at home, weakened team cohesion, and communication difficulties. These drawbacks can negatively affect organizational structure and reduce productivity if not properly addressed. The authors further emphasize that inadequate management of psycho-social risks in remote work environments—such as social isolation, work-life imbalance, and lack of managerial support—can lead to increased absenteeism, higher turnover rates, and lower employee well-being, ultimately undermining organizational performance.

#### Spatial factors, relocation practices and mobility patterns

##### Division of hours between remote and in-office work

Transitioning from the broader benefits, challenges, and implications of remote work to the specific spatial dynamics of participants’ working arrangements, respondents were asked to quantify their weekly division of hours between remote and in-office work through the item: “On average, what amount of time of your weekly work schedule do you perform remotely/ in the office?”.

Participants selected their preferred location for remote work from three options: (1) the home, (2) a third place (libraries, cafes, community buildings, etc.) and (3) a co-working space. For those preferring third places or co-working spaces, they were asked to indicate by choosing yes or no if their preference was linked to (1) the availability of car parking, (2) the accessibility via public transport and (3) its proximity within walking or cycling distance from home. This section aimed to provide an understanding of participants’ remote work arrangements.

##### Access to services and amenities

The survey assessed access to services and amenities through the item: “When working remotely, do you currently have access to the following amenities within a 15-minute walk?”^48,49^. Participants were also asked to indicate the importance of each amenity. This approach enables an evaluation of the accessibility to essential amenities for remote workers, linking remote work preferences to daily routines and lifestyle choices. Proximity to amenities such as schools, groceries, green spaces and healthcare services can influence the attractiveness and feasibility of remote work, shaping work-life balance, productivity, and overall well-being. Research suggests that remote workers increasingly prioritize access to amenities that enhance their quality of life and work-life balance, while the shift towards remote work has led to a revaluation of location attractiveness, with emphasis on green spaces, educational facilities, and social amenities^50^. These trends underscore the growing importance of localized infrastructure in supporting the evolving needs of workers.

To assess access to services and amenities during remote work, participants evaluated a predefined list comprising of (1) childcare/schools, (2) restaurants/cafes, (3) grocery store/ retail (e.g., laundry, stationery shops, etc.), (4) park/green spaces, (5) healthcare facilities, (6) leisure activities (e.g., cinemas, theatres, shopping), (7) sports facilities and (8) public transport connections. For each item, participants were asked to indicate both availability within a 15-minute walk and its significance by selecting one of four options: (1) yes, and it is important; (2) yes, but it is not important; (3) no, but it would be important and (4) no, but it is not important. This approach provided insights into the accessibility and perceived value of various amenities within walking distance for remote workers.

##### Changes in workplace and/or place of living

The survey explored relocation behavior through the item: “Have you changed your place of living and/or working due to favorable remote working arrangements since 2020?” and “Name the 5 most important reasons that led to your place of living relocation”.

Decisions to relocate in the context of remote work are influenced by a variety of factors, including personal preferences, family ties, financial capacity, and connections within the community. With the rise of remote work, employees are placing greater emphasis on factors other than workplace proximity, resulting in changes to residential trends. Without the constraints of daily commuting, many individuals choose to relocate to more appealing areas or pursue affordable housing options in suburban or rural regions as well as smaller cities^8,19,51^. At the same time, the decoupling of living and working locations has created a new market in which localities compete for the physical presence of remote workers^52^. This trend could potentially bridge the gap between the urban – rural divide fostering a more equitable distribution of population and resources. However, it may also exacerbate urban sprawl, posing challenges for sustainable regional development^51^. These dynamics highlight the complex interplay between remote work, residential mobility and broader spatial planning considerations. As a result, strategies such as relocation incentives and the creation of coworking spaces have gained prominence, supported by initiatives from both public authorities and private enterprises^52,53^.

In specific, housing preferences are influenced by several factors including the availability of neighborhood amenities, the quality of local schools, environmental conditions such as pollution and the demands of daily, inflexible commutes. However, in the context of remote working, the absence of a fixed commuting requirement, especially to work, allows individuals greater freedom to live and work from everywhere.

To explore changes in workplace and residence because of remote work, participants were first asked whether they had changed their geographic place of work since 2020. If they responded affirmatively, they were prompted to specify their previous work country and provide the corresponding postal code. Similarly, participants were asked whether they had changed their place of living due to favourable remote working arrangements since 2020. Predefined response options included (1) no, (2) yes, I changed it once, and (3) yes, I changed it regularly. Respondents selecting options 2 or 3 were subsequently asked to specify their previous country of residence and postal code. These questions allowed a comparative analysis of participants’ past and current places of living, hinting at relocation trends linked to remote work.

For respondents who indicated they had relocated since 2020 (options 2 or 3), the final question aimed to identify the key factors influencing their decision to move due to remote work. Participants were presented with a list of 19 potential reasons and were asked to select up to five significant ones in determining their current place of residence. The options included: (1) affordable housing options, (2) living expenses, (3) housing size, (4) change in workplace (different employer, enterprise, etc.), (5) change in employee services, (6) retirement, (7) schooling for children, (8) health reasons, (9) proximity to workplace area / co-working spaces, (10) working conditions environment, (11) proximity to nature, (12) quality of life, (13) safety, (14) digital infrastructure, (15) proximity to family and friends, (16) proximity to urban area, (17) proximity to rural area, (18) cultural immersion, (19) access to like-minded community (remote workers, digital nomads). This question allowed us to capture the different motivations behind relocation.

#### Commuting patterns

The survey explored commuting behaviour through the item: “How much time do you spend on commuting to work and how do you usually commute to work?”. The impact of remote work on transportation and mobility patterns is a key debate, as the shift towards remote and hybrid work arrangements has redefined traditional commuting demand. Peak travel days and hours are shifting complicating the prediction of travel behaviours and the strategic planning of infrastructure investments. Research indicates that remote work can contribute to the reduction of daily car usage, alleviating traffic congestion, and promoting the use of eco-friendly means of transportation. However, it is also indicated that remote work might lead to longer, but less frequent commutes, altering mobility dynamics^54,55^. The trend of remote workers to relocate to suburban areas or smaller cities further reshapes transportation needs. Such relocations may increase pressure on existing road infrastructure and create demand for expanded public transportation systems, potential leading to greater reliance on private vehicles^8^. Additionally, the travel behaviour of remote workers is significantly influenced by the surrounding land uses and allocated amenities, as well as the accessibility of transportation network near their residences^55^. These findings highlight the complex interplay between remote work, mobility patterns and urban planning, necessitating adaptive approaches to infrastructure and transportation system development.

To assess commuting patterns, we asked participants to report on the average time spent commuting to work, measured in minutes per commute. This provided a foundation for conceptualising the extent of their commuting range. Participants were then asked to indicate all applicable modes of transport they used from a predefined list: (1) on foot, (2) bicycle, (3) scooter/motorcycle, (4) public transport (bus, metro, tram, ferry, train), (5) personal car, (6) shared car, or (7) not applicable for those that do not commute for work (“other” category). These questions were designed to capture the diversity of commuting behaviours in relation to the ways remote work might influence or be influenced by commuting patterns.

#### Demographics and employment context

The last section of the survey focuses on collecting demographic information to contextualize participants’ responses within a broader socioeconomic context and professional framework. Participants were asked to provide details on their gender, age, years of education and professional experience. Furthermore, the survey collected information on participant’s current place of residence and work, including geographical location (municipality), as well as the industry sector in which they are employed, categorized to the NACE industry classification. NACE (Nomenclature des Activités Économiques) is the European statistical classification of economic activities. It is a hierarchical system used to categorize and analyze economic activities across the European Union and beyond. NACE provides a framework for collecting and presenting statistical data related to economic activities in various domains like production, employment, and national accounts^56^. Employment status is also assessed, with subcategories ranging from paid employment and self-employment to voluntary work. This section concludes with an open-ended question, allowing participants to share any additional comments. These demographic insights ensure that the survey captures a diverse range of perspectives and enables the identification of patterns and trends across different demographics, professional groups and geographical context.

The European Union began to acknowledge economic, social and territorial cohesion as the foundational pillars of its overarching vision and developmental agenda. However, it was not until the mid-1980s that more concerted and formalized efforts were undertaken to advance these goals. In furtherance of these goals, the European Regional Development Fund, the European Social Fund Plus and the Cohesion Fund function as pivotal instruments, each target specific dimensions such as - innovation, environmental sustainability, transport, social inclusion- while converging upon the overarching goal of strengthening the EU’s territorial cohesion.

### Exclusion criteria

We do not exclude outliers. Participants are excluded if they meet at least one of the following criteria: (1) Failure to give informed consent. (2) Taking the survey more than once, as indicated by Prolific ID; only the first test will be analyzed. (3) Failure to finish the survey.

## Data Records

The dataset is available on the Open Science Framework (OSF)^57^, including an additional data repository at: https://osf.io/d32u7/files/osfstorage#.

This repository includes the survey questionnaire, which is provided as a separate Word file. All figures referenced in the manuscript are available in the corresponding figure folder.

The primary dataset is provided in the file named “RMAP_Data_Descriptor_Data.csv”. This file contains all raw survey responses collected as part of the RMAP study. It is structured as follows:

- Tab 1: “Data” – This tab contains the full dataset, with each row corresponding to an individual respondent and each column representing a survey variable or metadata field. Variables include respondent IDs, demographic data, remote work preferences, commuting behavior, spatial preferences, employment status, relocation reasons, and access to amenities, among others.
- Tab 2: “Variables” – This tab provides detailed descriptions for each variable included in the dataset. For each column in the data tab, the variable name, format (e.g., numeric, categorical, text), and a brief explanation of the survey question or coding scheme are documented. This includes clarification of Likert scale codings, multi-response variables (e.g., commuting modes), and derived typology variables (e.g., typology_current_place_of_work).

The file structure supports reproducibility and transparency. For ease of use, all variable names in the dataset follow a consistent naming convention based on the original questionnaire items, which allows for clear tracing back to the original survey content. Complex items such as multi-part questions or questions with follow-up conditions are flattened into columnar format with consistent labeling. Variables are anonymized, and no personally identifying information is collected or stored. Free-text responses are not included in this release to ensure participant confidentiality. Further metadata, including coding decisions for categorical variables and derived fields, is provided in the “Variables” tab. This structure enables researchers to readily understand and work with the dataset, ensuring clarity in both its content and intended use.

### Data overview

#### Sample representativeness and generalizability

To assess the generalizability of our findings, we compared the demographic profile of our sample (n = 20,013) with European Union benchmarks using Eurostat data. As noted earlier, the survey was conducted via Prolific using a stratified sampling approach, which aimed to ensure gender parity and an age distribution representative of EU averages. However, we focus only on remote workers. As a result, the sample achieved a relatively balanced distribution in terms of gender and age.

However, the dataset shows an overrepresentation of individuals with higher education—approximately 62% of respondents—compared to the EU-27 average of about 32% (Eurostat, 2023). Additionally, there is a higher concentration of participants working in sectors such as information technology, research, and education. These trends are consistent with patterns frequently observed in online, self-selected survey samples, where participation often skews toward more highly educated and digitally connected individuals.

Accordingly, we advise caution in generalizing the results to the broader population, particularly to underrepresented groups with lower levels of education or limited digital access. Nonetheless, the large sample size and broad geographic coverage across Europe enable meaningful insights into remote work trends across urban and rural settings.

Further detail on sample representativeness is provided in a supplementary document, where we present comparative figures illustrating the distribution of survey respondents alongside official population data from Eurostat. This includes key demographic and socioeconomic variables such as age, gender, industry sector, and education level.

In terms of age, both the sample and population distributions are approximately normal. However, since our study focuses on individuals currently in employment, the sample’s age distribution is more concentrated in the central working-age range, resulting in a sharper peak in the middle age brackets.

**Fig. 1:**
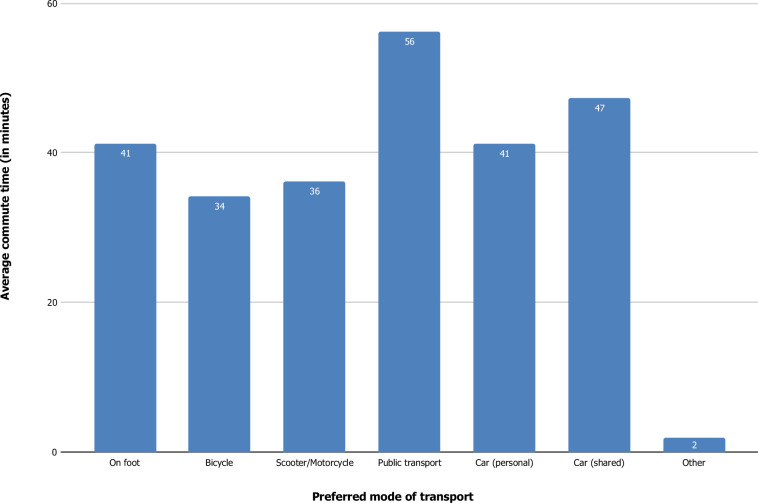
Average time spent for commuting to work. per mode of transport (in minutes) among the sample population (n = 20,013).

Figure 1 presents a bar chart depicting the average time respondents spend commuting to work based on their preferred mode of transportation. The data reveals that public transport involves the longest average commute time at 56.17 minutes, followed by shared cars (47.27 minutes) and personal cars (41.18 minutes). Walking (41.23 minutes), scooters/motorcycles (36.18 minutes), and bicycles (34.15 minutes) involve shorter commute times. The “other” category exhibits the lowest average commute time, at approximately 1.96 minutes.

**Fig. 2:**
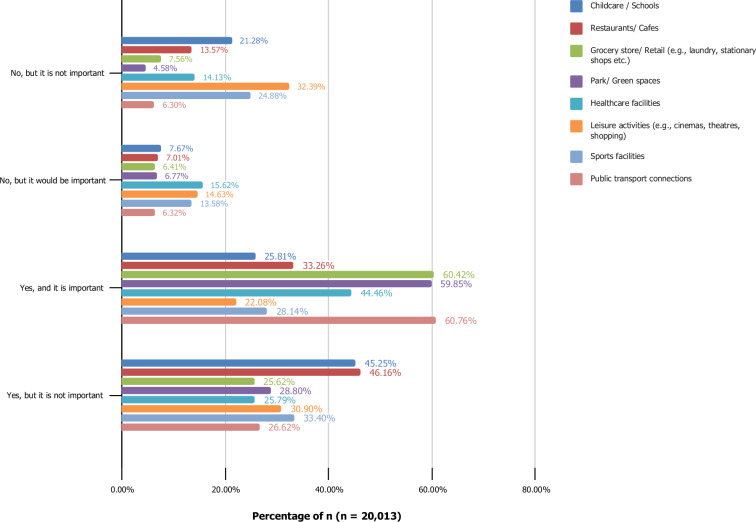
Respondents’ access to and perceived importance of amenities within a 15-minute walking distance (n = 20,013).

Figure 2 illustrates the distribution of respondents based on their access to various amenities within a 15-minute walking distance and the importance they place on these amenities. The data is grouped into four categories for each amenity: “Yes, and it is important,” “Yes, but it is not important,” “No, but it would be important,” and “No, but it is not important.” Amenities include public transport connections, sports facilities, leisure activities (e.g., cinemas, theaters, shopping), healthcare facilities, parks/green spaces, grocery stores/retail, restaurants/cafes, and childcare/schools.

The majority of respondents indicated that grocery stores/retail are both accessible and important (12,091 individuals), followed closely by public transport connections (12,160 individuals) and leisure activities (11,978 individuals). Parks/green spaces are accessible and important for 8,897 individuals. In contrast, sports facilities and healthcare facilities show higher proportions of respondents lacking access but finding them important (2,717 and 3,127 individuals, respectively). Childcare/schools have the smallest proportion of respondents who find them both accessible and important (5,165 individuals).

## Technical Validation

The technical validation aimed to assess the quality, structure, and consistency of the dataset, particularly in relation to the key constructs measured through the survey responses on remote work and work-life balance. This process involved several steps: (i) data preprocessing, including handling missing values, recoding categorical variables, and identifying outliers; (ii) selection and preparation of 8 Likert-scale items for further analysis; (iii) assessment of dataset suitability and dimensionality reduction using Exploratory Factor Analysis (EFA); (iv) computation of aggregate factor scores; and (v) validation of factor score robustness through comparisons across demographic subgroups using Bayesian ANOVA and effect size estimation. The following paragraphs detail these steps.

We have included a summary of missing data as part of the representativeness documentation, available as a separate file at: https://osf.io/d32u7/files/osfstorage/6874d91733d998de9a2f959d.

The variables included in the dataset contain nominal, ordinal and numeric characteristics, and the nominal and ordinal characteristics were recoded into a numeric form in support of statistical analysis. Participants are based on the sampling procedure and were opportunistically recruited; hence it is not expected that the sample has an equal distribution across gender and age ranges. Rather, the sample could be recognised as a large convenience sample measuring trends on remote working across heterogeneous individuals.

Initially, the missing values of the dataset were tabulated and the presence of outliers was investigated with the 1.5 times the IQR criterion.

A subset of eight items (ranked on a five-point Likert scale) related to remote work and work-life balance was selected from the dataset to undergo exploratory factor analysis (EFA). EFA was chosen because it is an inductive, data-driven method that helps uncover underlying patterns and reduce the complexity of interrelated variables. More specifically, this approach enabled the transformation of multiple correlated survey items into a reduced set of meaningful dimensions, allowing for a more interpretable assessment of respondents’ attitudes toward remote work and flexibility.

To assess the underlying structure of perceptions related to remote work, we conducted an EFA on the eight selected Likert-scale items. These items were specifically designed to capture key aspects of the remote work experience, including perceived productivity, work-life balance, social connectedness, autonomy, concentration, communication effectiveness, and overall satisfaction. As these items were newly developed for this study and had not been previously validated as a unified scale, EFA was used to determine whether a smaller number of latent dimensions could meaningfully summarize the data. This approach ensured that the items were not measuring entirely unrelated constructs and provided empirical justification for the creation of composite measures used in subsequent analyses.

The Likert-scale items included the following statements: 1. It is important for me to have the flexibility to choose my work location; 2. Remote work positively impacts my personal life; 3. It is important for me to adjust my work schedule based on personal circumstances; 4. I prefer remote work over in-office work; 5. Remote work negatively impacts my career advancement (promotion, recognition, skill development); 6. I find it difficult to maintain a healthy work-life balance while working remotely; 7. I find remote work more challenging than in-office work (e.g. isolation, more distractions, time zone differences); 8. I feel less productive while working remotely (more distractions, no focus, bad time management, etc.). The items were renamed systematically to facilitate interpretation. Prior to conducting factor analysis, the suitability of the dataset was assessed using the Kaiser-Meyer-Olkin (KMO) measure and Bartlett’s test of sphericity. The KMO measure evaluated the adequacy of the correlation matrix, with higher values indicating a greater appropriateness for factor analysis. Bartlett’s test was employed to determine whether the observed correlation matrix significantly deviated from an identity matrix, ensuring that sufficient inter-correlations existed among the variables.

To determine the most meaningful structure within the data, we identified the optimal number of factors as part of the exploratory factor analysis process, ensuring a balance between explanatory power and interpretability. To establish the optimal number of factors, a parallel analysis was conducted, comparing eigenvalues derived from the observed data against those obtained from randomly generated datasets. Based on these results, three factors were retained for further analysis. Factor extraction was performed using the minimum residual estimation method, with oblimin rotation applied to allow for correlated factors. Factor loadings were examined, and items were assigned to specific factors based on their highest loading values. The three factors identified through exploratory factor analysis were interpreted and labeled as follows: (1) *Work-Life Balance*, capturing respondents’ perceived ability to manage personal and professional responsibilities; (2) *Remote Work Productivity*, reflecting self-assessed efficiency and concentration while working remotely; and (3) *Social and Organizational Connectedness*, indicating the degree of social integration and connectedness to colleagues and the organization.

Following the identification of factor groupings, aggregated scale scores were computed for each factor by calculating the mean response across the corresponding Likert items. These scores provided composite measures representing underlying latent constructs. The computed factor scores were then integrated into the original dataset to facilitate further statistical analysis.

To assess the robustness of the factor structure and evaluate potential bias across demographic subgroups, we compared the aggregated factor scores across several background variables: gender, ethnicity, continent, country of residence, and age group. This step aimed to examine whether respondents from different sociodemographic backgrounds systematically differed in their perceptions of remote work, as reflected in the factor scores. Prior to analysis, the dataset was cleaned to retain only those subgroup levels with a sufficient number of observations, and all entries with missing factor scores were removed to ensure complete cases.

We conducted separate Bayesian ANOVA tests for each factor score to determine whether statistically meaningful differences existed across the demographic categories. Bayes Factors (BFs) were calculated for each comparison, using a prior scale adjustment to account for variability in group sizes and distributions. Given that Bayes Factors can be sensitive to sample size, we also reported omega squared (ω²) as an effect size measure to quantify the proportion of variance explained by the group differences^58^.

While some Bayes Factors suggested evidence for differences between groups, the consistently low effect sizes (ω² < 0.02) indicated that any observed differences were weak and of limited practical significance. These results suggest that the derived factor scores are relatively stable across key demographic subgroups, supporting the generalizability of our findings^59^.

One important limitation of our study concerns the scope of socioeconomic indicators included in the survey. Although variables such as household income and vehicle ownership would have allowed for a more detailed analysis of participants’ socioeconomic status, these were not included due to practical constraints. Given our large sample size and limited research budget, we prioritized survey brevity to reduce respondent fatigue and ensure high-quality, complete responses. As a result, we relied on widely accepted proxy variables—namely, age, years of education, and professional experience—to capture key aspects of socioeconomic status. While this approach supports meaningful interpretation of patterns across social groups, we acknowledge that it provides only a partial view. Future studies with greater resources or narrower respondent targets could benefit from incorporating a broader set of indicators, including income and vehicle ownership, to allow for more nuanced analyses^60^.

## Supplementary information

Supplementary document

## Data Availability

No custom code was used. To better understand the drivers, enablers and barriers influencing the adoption of remote work, the project developed and conducted a large-scale survey covering multiple European countries.

## Data availability

The dataset is available on the Open Science Framework (OSF)^57^, including an additional data repository at: https://osf.io/d32u7/files/osfstorage#. We have included a summary of missing data as part of the representativeness documentation, available as a separate file at: https://osf.io/d32u7/files/osfstorage/6874d91733d998de9a2f959d.
