# Supplementary material for: A large-scale dataset for analysing remote working in urban and rural areas across Europe
Source: Sci Data. 2025 Oct 23;12:1681. doi: 10.1038/s41597-025-05972-z (PMC12549887; doi:10.1038/s41597-025-05972-z)
Supplement: Supplementary file 1 — Supplementary document [file 41597_2025_5972_MOESM1_ESM.docx]

**Dataset Representativeness**

Below we present a comparison between the distribution of our survey respondents and the corresponding population distribution across Europe, based on Eurostat data. The comparison includes key demographic and socioeconomic variables: age, gender, industry sector, and education level.

With regard to age, both distributions follow a roughly normal pattern. However, since our sample includes only individuals who are currently employed, the age distribution is more concentrated around the working-age population, resulting in a steeper peak in the middle age brackets.

Survey respondents by age:

Age distribution across Europe:

In terms of gender, like the even distribution of genders, we also see the survey respondents broadly divided equally between male and female respondents.

Survey respondents by gender:

Gender distribution across Europe:

In terms of sector-specific representation, much like employment distribution at the European level, we observe a high number of respondents from sectors including professional, scientific and technical activities, administrative and support service activities, public administration, defence, education, human health and social work activities; and medium number of respondents from sectors including manufacturing, construction, and arts, entertainment and recreation; other service activities; and limited respondents from sectors including agriculture, forestry and fishing, real estate activities. There is an overrepresentation of sectors, including information and communication, and financial and insurance activities, given their overrepresentation in remote working.

Survey respondents by sector:

Employment by sector across Europe:

Reviewer 2, comment 5; regarding missing values

Here is the percentage of missing values per survey question.

|  | **variable** | **pct_missing** | |  |
| --- | --- | --- | --- | --- |
|  |  | |  | |
| **1** | X.1 | | 0.0000000 | |
| **2** | Response.ID | | 0.0000000 | |
| **3** | Date.submitted | | 4.5756000 | |
| **4** | Start.language | | 0.0000000 | |
| **5** | Would.you.like.to.select.a.different.language. | | 0.2719595 | |
| **6** | Before.proceeding..we.would.like.to.kindly.draw.your.attention.to.the.following.information...CONTENTWe.aim.to.gather.valuable.insights.into.workers..perceptions..intentions..and.needs.related.to.remote.working.arrangements..Your.participation.is.crucial.in.shaping.our.understanding.of.the.evolving.landscape.of.remote.work..Thank.you.for.being.a.part.of.this.important.initiative...PARTICIPATIONYour.participation.in.this.survey.is.entirely.voluntary..and.you.have.the.right.to.refuse.or.discontinue.your.involvement.at.any.point..Please.note.that.completion.of.the.survey.is.necessary.to.receive.payment..and.any.incomplete.data.will.not.be.used.or.considered.in.the.analysis...BENEFITSParticipation.in.this.survey.is.compensated.only.for.those.who.receive.invitations.through.Prolific..If.you.access.the.survey.from.any.other.source..your.contribution.is.voluntary.and.without.payment..This.R.MAP.project.101132497.has.received.funding.from.the.European.Commission.to.pay.participants...RISKSParticipating.in.this.survey.carries.no.foreseeable.risks..By.completing.the.questionnaire..you.provide.consent.for.the.generated.data.to.be.used.for.research.and.its.associated.purposes..The.results.will.be.openly.disseminated.through.various.channels..including.scientific.publications.and.public.reports..ensuring.anonymity...ANONYMITYWe.do.not.gather.any.personally.identifying.information..As.a.result..your.responses.will.be.kept.anonymous...CONTACTFor.more.information.please.visit.https...r.map.eu.privacy.policy..If.you.have.questions.at.any.time.about.the.study.or.the.procedures..you.may.contact.us.viaemail.at.team.rim.eu.com..ELECTRONIC.CONSENTPlease.select.your.choice.below.Clicking.on.the..Agree..button.indicates.that...You.agree.to.the.above.information...You.are.clearly.informed...You.voluntarily.agree.to.participate...You.will.get.paid.only.if.invited.via.Prolific.AND.provide.complete.data...Your.anonymous.answers.can.be.used.for.research.and.exploitation.purposes...You.are.18.years.of.age.or.older. | | 0.5725464 | |
| **7** | Plebse.insert.your.Prolific.ID...chbnged.to.ensure.bnonymity. | | 0.9303879 | |
| **8** | Please.rate.the.following.statements.regarding.remote.work.on.a.scale.from..1..strongly.disagree.to..7..strongly.agree.......It.is.important.for.me.to.have.the.flexibility.to.choose.my.work.location.. | | 2.3522115 | |
| **9** | Please.rate.the.following.statements.regarding.remote.work.on.a.scale.from..1..strongly.disagree.to..7..strongly.agree.......Remote.work.positively.impacts.my.personal.life.. | | 2.3760676 | |
| **10** | Please.rate.the.following.statements.regarding.remote.work.on.a.scale.from..1..strongly.disagree.to..7..strongly.agree.......It.is.important.for.me.to.adjust.my.work.schedule.based.on.personal.circumstances.. | | 2.3856100 | |
| **11** | Please.rate.the.following.statements.regarding.remote.work.on.a.scale.from..1..strongly.disagree.to..7..strongly.agree.......I.prefer.remote.work.over.in.office.work.. | | 2.3856100 | |
| **12** | Please.rate.the.following.statements.regarding.remote.work.on.a.scale.from..1..strongly.disagree.to..7..strongly.agree.......Remote.work.negatively.impacts.my.career.advancement..promotion..recognition..skill.development... | | 2.3856100 | |
| **13** | Please.rate.the.following.statements.regarding.remote.work.on.a.scale.from..1..strongly.disagree.to..7..strongly.agree.......I.find.it.difficult.to.maintain.a.healthy.work.life.balance.while.working.remotely.. | | 2.3856100 | |
| **14** | Please.rate.the.following.statements.regarding.remote.work.on.a.scale.from..1..strongly.disagree.to..7..strongly.agree.......I.find.remote.work.more.challenging.than.in.office.work..e.g..isolation..more.distractions..time.zone.differences... | | 2.3856100 | |
| **15** | Please.rate.the.following.statements.regarding.remote.work.on.a.scale.from..1..strongly.disagree.to..7..strongly.agree.......I.feel.less.productive.while.working.remotely..more.distractions..no.focus..bad.time.management.etc.... | | 2.3856100 | |
| **16** | On.average..what.amount.of.time.of.your.weekly.work.schedule.do.you.perform.remotely..in.the.office.......Remotely...Hours. | | 6.9039553 | |
| **17** | On.average..what.amount.of.time.of.your.weekly.work.schedule.do.you.perform.remotely..in.the.office.......In.the.Office..Hours. | | 6.2168997 | |
| **18** | When.working.remotely..where.do.you.prefer.to.work.from.... | | 3.0583520 | |
| **19** | What.do.you.prefer.when.working.at.a.third.place.or.a.coworking.space...Availability.of.car.parking.. | | 94.1075433 | |
| **20** | What.do.you.prefer.when.working.at.a.third.place.or.a.coworking.space...Accessibility.via.public.transport.. | | 94.1075433 | |
| **21** | What.do.you.prefer.when.working.at.a.third.place.or.a.coworking.space...Proximity.within.walking.or.cycling.distance.from.home. | | 94.1075433 | |
| **22** | How.much.time.do.you.spend.on.commuting.to.work..in.minutes.per.commute.. | | 3.2730569 | |
| **23** | How.do.you.usually.commute.to.work..please.pick.all.that.apply....On.foot. | | 0.0000000 | |
| **24** | How.do.you.usually.commute.to.work..please.pick.all.that.apply....Bicycle. | | 0.0000000 | |
| **25** | How.do.you.usually.commute.to.work..please.pick.all.that.apply....Scooter.Motorcycle.. | | 0.0000000 | |
| **26** | How.do.you.usually.commute.to.work..please.pick.all.that.apply....Public.transport..Bus..Metro..Tram..Ferry..Train.. | | 0.0000000 | |
| **27** | How.do.you.usually.commute.to.work..please.pick.all.that.apply....Car..personal... | | 0.0000000 | |
| **28** | How.do.you.usually.commute.to.work..please.pick.all.that.apply....Car..shared.. | | 0.0000000 | |
| **29** | How.do.you.usually.commute.to.work..please.pick.all.that.apply....Not.applicable. | | 0.0000000 | |
| **30** | When.working.remotely..do.you.currently.have.access.to.the.following.amenities.within.a.15.minute.walk..If.so..please.indicate.whether.they.are.important.to.you....Childcare...Schools. | | 3.4543633 | |
| **31** | When.working.remotely..do.you.currently.have.access.to.the.following.amenities.within.a.15.minute.walk..If.so..please.indicate.whether.they.are.important.to.you....Restaurants..Cafes. | | 3.4543633 | |
| **32** | When.working.remotely..do.you.currently.have.access.to.the.following.amenities.within.a.15.minute.walk..If.so..please.indicate.whether.they.are.important.to.you....Grocery.store..Retail..e.g...laundry..stationary.shops.etc... | | 3.4543633 | |
| **33** | When.working.remotely..do.you.currently.have.access.to.the.following.amenities.within.a.15.minute.walk..If.so..please.indicate.whether.they.are.important.to.you....Park..Green.spaces. | | 3.4591345 | |
| **34** | When.working.remotely..do.you.currently.have.access.to.the.following.amenities.within.a.15.minute.walk..If.so..please.indicate.whether.they.are.important.to.you....Healthcare.facilities. | | 3.4543633 | |
| **35** | When.working.remotely..do.you.currently.have.access.to.the.following.amenities.within.a.15.minute.walk..If.so..please.indicate.whether.they.are.important.to.you....Leisure.activities..e.g...cinemas..theatres..shopping.. | | 3.4543633 | |
| **36** | When.working.remotely..do.you.currently.have.access.to.the.following.amenities.within.a.15.minute.walk..If.so..please.indicate.whether.they.are.important.to.you....Sports.facilities. | | 3.4543633 | |
| **37** | When.working.remotely..do.you.currently.have.access.to.the.following.amenities.within.a.15.minute.walk..If.so..please.indicate.whether.they.are.important.to.you....Public.transport.connections. | | 3.4543633 | |
| **38** | Did.you.change.your.geographic.place.of.work.since.2020..... | | 3.5020755 | |
| **39** | In.which.world.region.was.your.last.place.of.employment. | | 66.2531609 | |
| **40** | In.which.country.was.your.last.place.of.employment. | | 98.7356267 | |
| **41** | In.which.country.was.your.last.place.of.employment..1 | | 99.5419629 | |
| **42** | In.which.country.was.your.last.place.of.employment..2 | | 97.3710578 | |
| **43** | In.which.country.was.your.last.place.of.employment..3 | | 74.3308364 | |
| **44** | In.which.country.was.your.last.place.of.employment..4 | | 96.2927621 | |
| **45** | Please.provide.the.municipality.of.your.last.place.of.work..Please.be.as.specific.as.possible. | | 66.4535522 | |
| **46** | Have.you.changed.your.place.of.living.due.to.favourable.remote.working.arrangements.since.2020..... | | 3.7549501 | |
| **47** | In.which.world.region.was.your.last.place.of.living. | | 79.1736247 | |
| **48** | In.which.country.was.your.last.place.of.living. | | 99.4131399 | |
| **49** | In.which.country.was.your.last.place.of.living..1 | | 97.1372680 | |
| **50** | In.which.country.was.your.last.place.of.living..2 | | 97.2470061 | |
| **51** | In.which.country.was.your.last.place.of.living..3 | | 85.5575171 | |
| **52** | In.which.country.was.your.last.place.of.living..4 | | 99.8186936 | |
| **53** | Please.provide.the.municipality.of.your.last.place.of.living..Please.be.as.specific.as.possible. | | 79.2547354 | |
| **54** | Name.the.5.most.important.reasons.that.led.to.your.place.of.living.relocation.from.the.following.list...Affordable.housing.options.. | | 0.0000000 | |
| **55** | Name.the.5.most.important.reasons.that.led.to.your.place.of.living.relocation.from.the.following.list...Living.expenses. | | 0.0000000 | |
| **56** | Name.the.5.most.important.reasons.that.led.to.your.place.of.living.relocation.from.the.following.list...Housing.size. | | 0.0000000 | |
| **57** | Name.the.5.most.important.reasons.that.led.to.your.place.of.living.relocation.from.the.following.list...Change.in.Workplace..different.employer..enterprise..etc.... | | 0.0000000 | |
| **58** | Name.the.5.most.important.reasons.that.led.to.your.place.of.living.relocation.from.the.following.list...Change.in.employee.services. | | 0.0000000 | |
| **59** | Name.the.5.most.important.reasons.that.led.to.your.place.of.living.relocation.from.the.following.list...Retirement. | | 0.0000000 | |
| **60** | Name.the.5.most.important.reasons.that.led.to.your.place.of.living.relocation.from.the.following.list...Schooling.for.children. | | 0.0000000 | |
| **61** | Name.the.5.most.important.reasons.that.led.to.your.place.of.living.relocation.from.the.following.list...Health.reasons. | | 0.0000000 | |
| **62** | Name.the.5.most.important.reasons.that.led.to.your.place.of.living.relocation.from.the.following.list...Proximity.to.workplace.area...co.working.spaces. | | 0.0000000 | |
| **63** | Name.the.5.most.important.reasons.that.led.to.your.place.of.living.relocation.from.the.following.list...Working.conditions.and.working.environment. | | 0.0000000 | |
| **64** | Name.the.5.most.important.reasons.that.led.to.your.place.of.living.relocation.from.the.following.list...Proximity.to.nature.. | | 0.0000000 | |
| **65** | Name.the.5.most.important.reasons.that.led.to.your.place.of.living.relocation.from.the.following.list...Quality.of.life. | | 0.0000000 | |
| **66** | Name.the.5.most.important.reasons.that.led.to.your.place.of.living.relocation.from.the.following.list...Safety.. | | 0.0000000 | |
| **67** | Name.the.5.most.important.reasons.that.led.to.your.place.of.living.relocation.from.the.following.list...Digital.infrastructure.. | | 0.0000000 | |
| **68** | Name.the.5.most.important.reasons.that.led.to.your.place.of.living.relocation.from.the.following.list...Proximity.to.family.and.friends. | | 0.0000000 | |
| **69** | Name.the.5.most.important.reasons.that.led.to.your.place.of.living.relocation.from.the.following.list...Proximity.to.urban.area. | | 0.0000000 | |
| **70** | Name.the.5.most.important.reasons.that.led.to.your.place.of.living.relocation.from.the.following.list...Proximity.to.rural.area. | | 0.0000000 | |
| **71** | Name.the.5.most.important.reasons.that.led.to.your.place.of.living.relocation.from.the.following.list...Cultural.immersion. | | 0.0000000 | |
| **72** | Name.the.5.most.important.reasons.that.led.to.your.place.of.living.relocation.from.the.following.list...Access.to.a.like.minded.community..remote.workers..digital.nomads.. | | 0.0000000 | |
| **73** | Please.share.your.employment.status. | | 3.9171716 | |
| **74** | In.which.world.region.do.you.currently.work. | | 4.3274965 | |
| **75** | In.which.country.do.you.currently.work. | | 22.9209409 | |
| **76** | In.which.country.do.you.currently.work..1 | | 99.3081731 | |
| **77** | In.which.country.do.you.currently.work..2 | | 95.4625698 | |
| **78** | In.which.country.do.you.currently.work..3 | | 98.9407892 | |
| **79** | In.which.country.do.you.currently.work..4 | | 87.7331934 | |
| **80** | Please.provide.the.municipality.of.your.current.place.of.work..Please.be.as.specific.as.possible. | | 4.2273009 | |
| **81** | Please.indicate.the.industry.sector.you.work.in. | | 6.5890548 | |
| **82** | In.which.world.region.do.you.currently.live. | | 4.2225297 | |
| **83** | In.which.country.do.you.currently.live. | | 22.7205496 | |
| **84** | In.which.country.do.you.currently.live..1 | | 95.4387137 | |
| **85** | In.which.country.do.you.currently.live..2 | | 99.3511141 | |
| **86** | In.which.country.do.you.currently.live..3 | | 98.9455604 | |
| **87** | In.which.country.do.you.currently.live..4 | | 87.7999905 | |
| **88** | Please.provide.the.municipality.of.your.current.place.of.living..Please.be.as.specific.as.possible. | | 4.4515483 | |
| **89** | What.is.your.gender. | | 4.4181497 | |
| **90** | Please.share.the.following...Your.age.in.years.. | | 4.5088029 | |
| **91** | Please.share.the.following...Total.years.of.full.time.education..from.primary.school.to.higher.education... | | 4.7855337 | |
| **92** | Please.share.the.following...Total.years.of.professional.working.life..count.part.time.work.as.a.fraction..e.g...0.5.for.half.time.work... | | 13.1113126 | |
| **93** | X | | 64.5402930 | |
| **94** | Remote.Office.Work | | 1.0401260 | |
| **95** | Ethnicity.Simplified | | 1.0401260 | |
| **96** | Country.of.Residence | | 1.0401260 | |
| **97** | Nationality | | 1.0401260 | |
| **98** | Student.Status | | 1.0401260 | |
| **99** | Employment.Status | | 1.0401260 | |
| **100** | last_place_of_work | | 79.3740159 | |
| **101** | country_of_last_place_of_work | | 79.3692447 | |
| **102** | typology_last_place_of_work | | 79.3692447 | |
| **103** | last_place_of_living | | 86.8075767 | |
| **104** | country_of_last_place_of_living | | 86.8075767 | |
| **105** | typology_last_place_of_living | | 86.8171191 | |
| **106** | current_place_of_work | | 36.2278735 | |
| **107** | country_of_current_place_of_work | | 36.2278735 | |
| **108** | typology_current_place_of_work | | 36.2278735 | |
| **109** | current_place_of_living | | 48.7904957 | |
| **110** | country_of_current_place_of_living | | 48.7761821 | |
| **111** | typology_current_place_of_living | | 48.7761821 | |
